# Effect of Carvedilol on Reduction in Heart Rate in Patients With Chronic Atrial Fibrillation

**DOI:** 10.4021/jocmr1581w

**Published:** 2013-10-12

**Authors:** Eitaro Kodani, Shin Matsumoto, Osamu Igawa, Yoshiki Kusama, Hirotsugu Atarashi

**Affiliations:** a Department of Internal Medicine and Cardiology, Nippon Medical School Tama-Nagayama Hospital, Tokyo, Japan

**Keywords:** Atrial fibrillation, Carvedilol, Heart rate, Total heart beat, AFQLQ

## Abstract

**Background:**

Currently, β-blockers are used most frequently for the purpose of heart rate (HR) control in patients with atrial fibrillation (AF) in worldwide. Carvedilol is one of common β-blockers and known to be effective for hypertension and heart failure. However, little can be found the information about the HR-lowering effect of carvedilol in patients with AF without heart failure. Therefore, we conducted this study to investigate the effect of carvedilol on HR in 3-minute electrocardiogram (ECG) and total heart beats (THBs) in 24-hour Holter ECG monitoring in patients with persistent or permanent AF.

**Methods:**

A total of 13 hypertensive patients (73 ± 12 years, 7 males) with AF and HR 90 bpm or more were enrolled. All patients received carvedilol from 5 mg/day. The dose of drug was titrated every 4 weeks and raised to 10 or 20 mg/day if HR was 80 bpm or more.

**Results:**

Mean HR was decreased from 101.9 ± 13.9 to 85.2 ± 15.2 bpm (P < 0.05) after treatment with carvedilol. THBs were also significantly decreased from 128 to 115 × 1,000/day (P < 0.001). Percent reduction in HR and THBs were 13.9% and 10.7%, respectively. The scores of Atrial Fibrillation Quality of Life Questionnaire (AFQLQ) did not change. Only one patient was required to discontinue carvedilol due to congestive heart failure.

**Conclusions:**

We observed that carvedilol certainly reduced HR in patients with chronic AF. We believe that the effect of carvedilol on the reduction in HR can contribute to the management of AF patients treated with rate-control strategy.

## Introduction

Atrial fibrillation (AF) is a most common sustained arrhythmia in daily clinical practice. Since AF is a potent risk factor for disability and impairing quality of life (QOL) associated with cardiogenic ischemic stroke and congestive heart failure, AF is important disease to be managed. Current strategies for the management of AF include the medical treatment with antiarrhythmic drugs to maintain sinus rhythm or the use of rate-controlling drugs allowing AF to persist. In both approaches, the anticoagulation therapy should be recommended. According to the results from several large clinical trials after 2000 [1-5], the outcomes of both strategies with rhythm-control and rate-control were comparable for the long-term prognosis including mortality. For the rate-control strategy, digitals, a part of Ca channel blockers (CCBs), and β-adrenoreceptor blockers (β-blockers) have been recognized to be effective and used in clinical situation for a long time. In Atrial Fibrillation Follow-up Investigation of Rhythm Management (AFFIRM) study [2] for about 4,000 high-risk patients with AF conducted in the United States and Canada, β-blockers were used most frequently for heart rate control and shown to be most effective compared with the other rate-controlling drugs. In Japanese Rhythm Management Trial for Atrial Fibrillation (J-RHYTHM) study [5] for Japanese patients with AF treated in cardiology clinics and institutions, β-blockers were used most frequently for this purpose in Japan as well as in Western countries. In addition, in answers to questionnaires concerning “Therapeutic Guidance for Atrial Fibrillation” obtained from about 1,200 Japanese cardiologists in 2007 [6], β-blockers were most common agent for the rate-control strategy and selected by half of physicians as the first choice. Especially, carvedilol was prescribed frequently for both patients with and without heart failure. Treatment with β-blockers are recommended rather than digitals in AF patients preserved left ventricular function in “Guidelines for Pharmacotherapy of Atrial Fibrillation (JCS 2008)” [7, 8]. Although meta-analysis showed that the magnitude of heart rate reduction was significantly associated with survival benefit of β-blockers in patients with heart failure [9], there is lack of information about heart rate-lowering effect of carvedilol in patients with AF but without heart failure. Therefore, we conducted this study to investigate the effect of carvedilol on heart rate in a 3-minute electrocardiogram (ECG) and total heart beats using 24-hour Holter ECG monitoring in patients with chronic AF but without heart failure.

## Methods

### Patient population

A total of 13 patients with AF were enrolled in this study. All patients had been diagnosed hypertension and persistent or permanent AF prior to starting the carvedilol treatment. All patients exhibited heart rate of 90 beats/minute (bpm) or more evaluated by numbers of QRS complex during any one minute in 3-minute ECG; even though the patients had received any heart rate-lowering agents except β-blockers. Carvedilol was given to the patients who had no changes in their current medications for at least three months prior to starting carvedilol. Hypertension was diagnosed when office blood pressure was ≥ 140/90 mmHg [10], if the patient was taking medication for hypertension, or if the patient had a history of hypertension. Patients with history or currently treated congestive heart failure or receiving any β-blockers were excluded from this study. Patients with prior myocardial infarction within one month, surgery-related paroxysmal AF, Wolf-Parkinson-White syndrome, bronchial asthma, or pregnancy were also not eligible for this study. The enrolled patients were prohibited from changes in medications during observation period except in urgent situations. This study was approved by the Ethics Committee of Nippon Medical School Tama-Nagayama Hospital, and written informed consent was obtained from all patients.

### Treatment with carvedilol

All patients were given carvedilol starting from a dose of 5 mg/day. After observation for 4 - 8 weeks, the dose of carvedilol was raised to 10 mg/day if patient’s resting heart rate was 80 bpm or more [11, 12] and patient was able to continue this drug. Moreover after 8 - 12 weeks, the dose of carvedilol was increased to 20 mg/day if patient’s heart rate was still 80 bpm or more and patient had been well-tolerated. Then, patients were observed for 12 - 16 weeks after starting the treatment on acceptable final dose of carvedilol. Coadministration with the other heart rate-controlling drugs including digitals and CCBs was allowed if doses of these drugs were maintained through the study. In case of the development of adverse effect, discontinuation or reduction of carvedilol dose was permitted according to judgments by physicians.

### Measurements of heart rate and the other parameters

The primary endpoints of this study were 1) heart rate evaluated by numbers of QRS complex during any one minute in 3-minute ECG, 2) total heart beats in 24-hour Holter ECG monitoring before and after treatment with carvedilol. Secondary endpoints were patient QOL scores [13] and adverse effects. ECG was measured by CardioStar FCP-7431 (Fukuda Denshi Co., Ltd., Tokyo, Japan), and 24-hour Holter ECG monitoring was performed using FM-150 (Fukuda Denshi Co., Ltd., Tokyo, Japan). Patient QOL was evaluated at each observation period using the Japanese Society of Electrocardiology’s Atrial Fibrillation Quality of Life Questionnaire (AFQLQ), which comprises 3 subsets that include 26 questions concerning frequency of occurrence of 6 symptoms (palpitation, dizziness, shortness of breath, chest discomfort, irregular pulse, and pulse deficit) (AFQLQ1), the severity of these symptoms (AFQLQ2), and anxiety and limitation of daily activities related to AF and AF treatment (AFQLQ3). The scores of AFQLQ are totally 98 including 24, 18, and 56 in each subset, respectively, and higher score is better [13]. Office blood pressure and parameters in ECG including QT interval, heart rate-corrected QT interval (QTc) calculated by Bazett’s formula (= QT/RR^0.5^) [14], and width of QRS complex were also measured at each observation period. Holter ECG monitoring, echocardiography, and blood sampling were performed at before and after treatment with final dose of carvedilol. Data on the final doses of carvedilol were evaluated in case of adverse effect.

### Statistical analysis

Data are presented as the mean ± standard deviation (SD). Statistical differences of the parameters between at the baseline and at each observation period were analyzed by a one-way repeated-measures analysis of variance (ANOVA) followed by Student’s t-tests for paired data with the Bonferroni correction. Differences of the parameters between before and after treatment with carvedilol were analyzed by a paired Student’s t-test. Comparison among unrelated 2 or 3 groups were analyzed by Student’s t-test or one-way ANOVA. Differences with P-values of less than 0.05 were considered to be statistically significant. All statistical analyses were performed using the SigmaStat version 3.5 software program (Systat Software, Inc., Chicago, IL, USA).

## Results

### Patients’ characteristics

The baseline characteristics of patients in the present study are shown in Table 1. All patients had hypertension and chronic AF including the types of persistent in 84.6% and permanent in 15.4%. Only one patient (7.7%) had prior myocardial infarction. Seven patients (53.8%) had been treated for heart rate control with several medications such as digitalis in 6 patients (46.2%), including 2 cases with verapamil (15.4%), and bepridil in one (7.7%), as shown in Table 1. There were no significant differences in baseline characteristics among 3 groups according to the final daily doses of carvedilol. The baseline values of heart rate and the other parameters are shown in Tables 2-, 4.

**Table 1:** Characteristics of Patients

		Groups of final daily carvedilol doses		
	Overall	5 mg	10 mg	20 mg
Number of patients, n	13	4	3	6
Male gender, n (%)	7 (53.8)	3	0	4
Age, year (range)	73.2 ± 11.8 (41 - 86)	74.3 ± 10.8 (60 - 86)	80.7 ± 4.0 (76 - 83)	38.8 ± 14.2 (41 - 80)
Body height, cm	161.6 ± 12.9	168.0 ± 10.2	147.8 ± 5.4	164.1 ± 13.1
Body weight, kg	62.4 ± 18.1	57.4 ± 8.1	52.0 ± 2.2	70.9 ± 24.0
Body mass index, kg/m^2^	23.6 ± 4.7	20.4 ± 2.6	23.9 ± 2.2	25.7 ± 5.8
Duration of atrial fibrillation, year	7.4 ± 6.3	10.3 ± 9.2	2.8 ± 3.7	7.8 ± 3.8
Types of atrial fibrillation				
Persistent, n (%)	11 (84.6)	4	3	4
Permanent, n (%)	2 (15.4)	0	0	2
Underlying diseases				
Diabetes mellitus, n (%)	4 (30.8)	2	1	1
Dyslipidemia, n (%)	6 (46.2)	1	2	3
Prior myocardial infarction, n (%)	1 (7.7)	1	0	0
Coadministration				
Digitalis*, n (%)	6 (46.2)	2	1	3
Verapamil*, n (%)	2 (15.4)	0	0	2
Bepridil*, n (%)	1 (7.7)	0	0	1
Pilsicainide, n (%)	1 (7.7)	0	0	1
Warfarin, n (%)	11 (84.6)	3	3	5

* Heart rate-lowering drugs.

**Table 2:** Changes in Heart Rate After Treatment With Carvedilol

	Baseline	4 - 8 weeks	8 - 12 weeks	12 - 16 weeks	%change in heart rate after 12 - 16 weeks
Overall (N = 13)	101.9 ± 13.9	82.5 ± 12.6**	85.2 ± 10.4**	85.2 ± 15.2*	-13.9 ± 16.7
Dose of carvedilol, mg	-	5.0 ± 0.0	9.1 ± 2.0	13.1 ± 6.9	
Groups of final daily carvedilol doses					
5 mg (N = 4)	106.2 ± 19.4	75.2 ± 14.5*	72.3 ± 0.4	85.7 ± 27.4	-19.0 ± 19.2
10 mg (N = 3)	105.1 ± 12.6	75.6 ± 8.2	83.1 ± 6.9	79.6 ± 20.8	-16.3 ± 25.6
20 mg (N = 6)	97.5 ± 11.4	90.8 ± 8.8	90.5 ± 9.6*	87.9 ± 10.5	-9.4 ± 11.7
Usage of heart rate-lowering drugs					
Used (N = 7)	96.1 ± 7.4	85.6 ± 6.6*	82.5 ± 6.4*	91.3 ± 13.2	-4.4 ± 13.4^†^
Unused (N = 6)	108.8 ± 7.1	78.9 ± 17.3*	88.4 ± 13.9*	77.9 ± 15.3*	-25.1 ± 13.3

Data are means ± SD, bpm. ** P < 0.01, * P < 0.05 vs. Baseline, ^†^ P < 0.05 vs. Unused.

**Table 3:** Changes in Parameters of 24-Hour Holter Electrocardiogram Monitoring

	Baseline	12 - 16 weeks	%change in total heart beats
Overall (N = 11)			
Total heart beats, /day	128,411 ± 12,690	114,526 ± 11580***	-10.7 ± 4.5
Maximum heart rate, bpm	164 ± 8	134 ± 12***	
Minimum heart rate, bpm	60 ± 9	56 ± 7	
Mean heart rate, bpm	93 ± 12	82 ± 8***	
Groups of final daily carvedilol doses			
5 mg (N = 2)			
Total heart beats, /day	131,382 ± 14,551	113,070 ± 5,218	-13.6 ± 5.6
Maximum heart rate, bpm	159 ± 11	144 ± 11	
Minimum heart rate, bpm	58 ± 20	50 ± 2	
Mean heart rate, bpm	97 ± 8	82 ± 4	
10 mg (N = 3)			
Total heart beats, /day	119,794 ± 15,193	104,539 ± 8,313*	-12.4 ± 4.4
Maximum heart rate, bpm	162 ± 12	121 ± 14*	
Minimum heart rate, bpm	54 ± 11	51 ± 7	
Mean heart rate, bpm	88 ± 9	75 ± 4*	
20 mg (N = 6)			
Total heart beats, /day	131,730 ± 11,229	120,005 ± 11,763**	-8.9 ± 4.1
Maximum heart rate, bpm	167 ± 7	137 ± 5***	
Minimum heart rate, bpm	63 ± 5	60 ± 6	
Mean heart rate, bpm	95 ± 8	85 ± 4**	

Data are means ± SD. *** P < 0.001, ** P < 0.01, * P < 0.05 vs. Baseline.

**Table 4:** Changes in AFQLQ Scores and Other Parameters

	Baseline	4 - 8 weeks	8 - 12 weeks	12 - 16 weeks
AFQLQ (maximum points = 98)	84 ± 11	85 ± 7	81 ± 11	86 ± 9
AFQLQ1 (24)	18 ± 5	17 ± 5	17 ± 5	19 ± 5
AFQLQ2 (18)	15 ± 3	15 ± 2	15 ± 3	16 ± 2
AFQLQ3 (56)	51 ± 5	53 ± 3	50 ± 6	52 ± 5
Office blood pressure				
Systolic blood pressure, mmHg	123.5 ± 9.4	121.0 ± 13.2	127.6 ± 6.6	124.0 ± 11.8
Diastolic blood pressure, mmHg	75.1 ± 8.2	73.8 ± 6.4	73.1 ± 7.4	74.9 ± 6.6
Electrocardiogram				
Heat beats for 3 min, /3 min	306 ± 41	248 ± 38**	256 ± 31**	252 ± 42*
QT interval, msec	333 ± 24	368 ± 31**	359 ± 21**	367 ± 31*
QTc interval, msec	437 ± 20	437 ± 25	435 ± 26	440 ± 30
Width of QRS, msec	91 ± 8	91 ± 8	91 ± 9	93 ± 9
Echocardiogram				
LAD, mm	41.1 ± 4.6	-	-	44.1 ± 4.9*
LVDd, mm	44.4 ± 4.4	-	-	46.8 ± 3.3
LVDs, mm	30.0 ± 3.1	-	-	32.2 ± 2.6*
%FS, %	32.4 ± 4.2	-	-	31.8 ± 4.9
LVEF, %	60.3 ± 6.2	-	-	58.9 ± 7.4
Blood Examinations				
BNP, pg/mL	134 ± 101	-	-	166 ± 108
hs-CRP, mg/dL	0.100 ± 0.144	-	-	0.058 ± 0.038

QTc: heart rate-corrected QT (= QT/RR0.5 by Bazett’s formula); AFQLQ: Atrial Fibrillation Quality of Life Questionnaire; LAD: left atrial dimension; LVDd: left ventricular end-diastolic dimension; LVDs: left ventricular end-systolic dimension; %FS: %change in fractioning shortening; LVEF: left ventricular ejection fraction (Teichholz); BNP: brain natriuretic peptide; hsCRP: high-sensitivity C-reactive protein. Data are means ± SD. ** P < 0.01, * P < 0.05 vs. Baseline.

### Changes in heart rate in 3-minute ECG

The changes in heart rate at each observation period are shown in Table 2. The mean values of heart rate in all patients were decreased from 101.9 ± 13.9 to 85.2 ± 15.2 bpm (P < 0.05). Percent reduction in heart rate was 13.9% on final doses of carvedilol. Dividing the patients into 3 groups according to the final daily doses of carvedilol, %reduction in heart rate in the group of 5 mg was largest followed by the groups of 10 mg and 20 mg, but not statistically significant. Whereas, %reduction in heart rate in patients without the use of heart rate-lowering drugs was significantly larger than that in those with use of these drugs (P = 0.018) (Table 2).

### Changes in total heart beats in 24-hour Holer ECG monitoring

The results of Holter ECG monitoring were shown in Table 3. Total heart beats were significantly decreased from 128 to 115 thousands/day (P < 0.001). Percent reduction in total heart beats was 10.7%. The reduction of total heart beasts was observed consistently in all subjects. In addition, values of maximum and mean heart rate were also significantly decreased after treatment with carvedilol, but the changes in minimum heart rate were not significant (Table 3).

### Changes in the scores of AFQLQ

The scores of AFQLQ including either 3 subset (AFQLQ1, AFQLQ2, or AFQLQ3) did not improve after treatment with carvedilol in patients with chronic AF (Table 4).

### Adverse effects

Adverse effects were observed in 3 cases. Of these, only one male patient was required to discontinue carvedilol 8 weeks after treatment with 5 mg/day due to the occurrence of congestive heart failure. Although his left ventricular function was preserved at 58% in ejection fraction, he was the sole patient suffered from old myocardial infarction and the oldest (86 years) in this study. One patient felt tinnitus during receiving carvedilol but he was able to complete the protocol with the dose of 20 mg/day. In another female patient, skin eruption appeared during observation period which was diagnosed asteatotic eczema, not an allergy for carvedilol. She could finish the protocol on her final dose of 10 mg/day. All adverse effects were not severe and the latter two incidents were probably not associated with carvedilol. One patient could not complete follow-up period except adverse effect because AF returned to sinus rhythm after finishing evaluation of parameters on the dose of 5 mg/day.

### Changes in the other parameters

Changes in the other parameters measured in the present study were summarized in Table 4. Although carvedilol is originally the drug for lowering blood pressure, either systolic or diastolic office blood pressure did not change significantly, because the blood pressures were well-controlled before starting carvedilol. In the parameters of ECG, QT intervals were prolonged by carvedilol accompanied with the reduction in heart rate, but QTc intervals were not, because they were corrected by heart rate. In echocardiogram, the dimensions of left atrium and left ventricle were little larger than those before treatment. No significant effect on BNP or hs-CRP was observed in this small number of subjects (Table 4).

## Discussion

The major finding of the present study was that carvedilol significantly reduced heart rate in 3-minute ECG and total heart beats in 24-hour Holter ECG monitoring. Although this study was conducted in a small number of subjects, promising results for primary endpoints were observed.

### Pharmacological characteristics of carvedilol

Carvedilol, a nonselective β-blocker, has not only β-adrenoreceptor blocking effect but also vasodilating effect resulting in the reduction in total peripheral vascular resistance. The latter is mainly thought to be caused by α_1_-adrenoreceptor blocking action [15]. In addition, as several investigators reported, carvedilol has antioxidative effect [16-18] and a potential of clinical benefit for not only congestive heart failure [19-22] but also other comorbid conditions, including coronary artery disease, stroke, hypertension, renal failure, diabetes, and AF, that can independently contribute to the progression of heart failure [17, 23].

### Effect of carvedilol on heart rate in patients with chronic AF

Even in stable outpatients with chronic AF, uncontrolled rapid heart rate can result in the development of congestive heart failure and/or impairment of QOL. Therefore, heart rate control is important and necessary to prevent heart failure and any impairment, though mortality in patients treated with rate-control strategy was comparable with those in rhythm-control strategy [1-5].

Present study demonstrated that carvedilol could certainly reduce heart rate in patients with chronic AF. However, %change in heart rate was not consistent in all patients. In fact, %reduction in heart rate in the groups of lower doses of carvedilol was larger than that in the group of 20 mg, despite it did not reach significant difference. The reason for these results could be explained by that patients were not randomized to divide into 3 groups in this study and the final daily dose of carvedilol was chosen by titration according to heart rate before raising a dose and tolerance for this drug. Similarly, %reduction in heart rate in patients with other heart rate-lowering drugs was significant smaller than that without use of these drugs. These results indicated that the patients consequently needed higher doses of carvedilol and/or those with the use of other heart rate-lowering drugs were resistant to the heart rate-lowering therapy. The sympathetic nerve activity in these patients would have been higher.

### Effect of carvedilol on total heart beats in patients with chronic AF

Since the casual heart rate can easily be modified by unstable situation, in particular the status of sympathetic nerve activity as well as white coat phenomenon on office blood pressure, total heart beats in 24-hour Holter ECG monitoring were evaluated to confirm the heart rate-lowering effect of carvedilol. Present study demonstrated that carvedilol could certainly reduce total heart beats in 24-hour Holter ECG monitoring in patients with chronic AF. An interesting finding was that carvedilol did not affect minimum heart rate despite maximum and mean heart rates significantly decreased; suggesting that carvedilol would not cause excessive response resulting in bradycardia. It would be beneficial for the management of AF patients to avoid any complication caused by bradycardia such as pacemaker implantation. Another important finding that total heart beats and mean heart rate in 24-hour Holter ECG were definitely decreased by the treatment with carvedilol in all subjects; even in some patients with increasing response to casual heart rate in the situation of outpatient clinic; indicating that the effect of heart rate-controlling drugs should be evaluated by not only casual heart rate but also total heart beats or mean heart rate using 24-hour Holter ECG monitoring.

### Effect of carvedilol on AFQLQ

No improvement of AFQLQ scores could be found after treatment with carvedilol in the present study. In J-RHYTHM study [5], the AFQLQ scores improved in both rhythm-control and rate-control strategies during follow-up period for 30 months which was longer than that in our protocol. All subjects in J-RHYTHM study were patients with paroxysmal AF and only AFQLQ1 subset scores were higher in patients managed with rhythm-control than those in rate-control strategy [5]. However, all subjects in the present study were patients with persistent or permanent AF. Therefore, the AFQLQ scores in all 3 subsets at baseline were higher than those observed in J-RHYTHM study [5].

### Adverse effects of carvedilol

It was required to discontinue carvedilol in only one case with old myocardial infarction. This fact taught us again that β-blockers should be prescribed with caution to elderly patients even though who have no history of heart failure and ejection fraction is within normal range.

### Study limitations

This study had several limitations. First, this study was performed in a single institution and had a small sample size. It was actually difficult to recruit patients for this study, because most AF patients had already received any β-blocker and their heart rates were well-controlled less than 90 bpm which was enroll criteria. However, even in a small number of subjects, the heart rate-lowering effect of carvedilol was obvious and statistically significant. Second, the dose of carvedilol was not randomized. Since the final daily doses of carvedilol were determined by titration, it could be explained the reason why %reduction in heart rate in patients receiving maximum dose of carvedilol was less than that in those receiving lower doses. However, the present method of titration to raise a dose of β-blocker would be more safety and recommended in real-world clinical situation.

In conclusion, we observed that administration of carvedilol reduced heart rate in patients with chronic AF without congestive heart failure. We believe that the effect of carvedilol on the reduction in heart rate can contribute to the management of AF patients treated with rate-control strategy and the prevention from complications in future.

## Declaration

The summary of this study was presented in the 60th Annual Scientific Session of the Japanese College of Cardiology, in Kanazawa, Japan, on Sept 15, 2012.

## References

[1] Hohnloser SH, Kuck KH, Lilienthal J (2000). Rhythm or rate control in atrial fibrillation--Pharmacological Intervention in Atrial Fibrillation (PIAF): a randomised trial. Lancet.

[2] Wyse DG, Waldo AL, DiMarco JP, Domanski MJ, Rosenberg Y, Schron EB, Kellen JC (2002). A comparison of rate control and rhythm control in patients with atrial fibrillation. N Engl J Med.

[3] Van Gelder IC, Hagens VE, Bosker HA, Kingma JH, Kamp O, Kingma T, Said SA (2002). A comparison of rate control and rhythm control in patients with recurrent persistent atrial fibrillation. N Engl J Med.

[4] Carlsson J, Miketic S, Windeler J, Cuneo A, Haun S, Micus S, Walter S (2003). Randomized trial of rate-control versus rhythm-control in persistent atrial fibrillation: the Strategies of Treatment of Atrial Fibrillation (STAF) study. J Am Coll Cardiol.

[5] Ogawa S, Yamashita T, Yamazaki T, Aizawa Y, Atarashi H, Inoue H, Ohe T (2009). Optimal treatment strategy for patients with paroxysmal atrial fibrillation: J-RHYTHM Study. Circ J.

[6] Atarashi H (2008). Answers to Questionnaires Concerning "Therapeutic Guidance for Atrial Fibrillation". Prog Med.

[7] Ogawa S, Aizawa Y, Atarashi H, Inoue H, Okumura K, Kamakura S, Kumagai K (2008). Guidelines for Pharmacotherapy of Atrial Fibrillation (JCS 2008). Circ J.

[8] (2010). Guidelines for pharmacotherapy of atrial fibrillation (JCS 2008): digest version. Circ J.

[9] McAlister FA, Wiebe N, Ezekowitz JA, Leung AA, Armstrong PW (2009). Meta-analysis: beta-blocker dose, heart rate reduction, and death in patients with heart failure. Ann Intern Med.

[10] Chobanian AV, Bakris GL, Black HR, Cushman WC, Green LA, Izzo JL, Jr, Jones DW (2003). Seventh report of the Joint National Committee on Prevention, Detection, Evaluation, and Treatment of High Blood Pressure. Hypertension.

[11] Van Gelder IC, Van Veldhuisen DJ, Crijns HJ, Tuininga YS, Tijssen JG, Alings AM, Bosker HA (2006). RAte Control Efficacy in permanent atrial fibrillation: a comparison between lenient versus strict rate control in patients with and without heart failure. Background, aims, and design of RACE II. Am Heart J.

[12] Fuster V, Ryden LE, Asinger RW, Cannom DS, Crijns HJ, Frye RL, Halperin JL (2001). ACC/AHA/ESC guidelines for the management of patients with atrial fibrillation: executive summary. A Report of the American College of Cardiology/ American Heart Association Task Force on Practice Guidelines and the European Society of Cardiology Committee for Practice Guidelines and Policy Conferences (Committee to Develop Guidelines for the Management of Patients With Atrial Fibrillation): developed in Collaboration With the North American Society of Pacing and Electrophysiology. J Am Coll Cardiol.

[13] Yamashita T, Kumagai K, Koretsune Y, Mitamura H, Okumura K, Ogawa S, Naito K (2003). A new method for evaluating quality of life specific to patients with atrial fibrillation: Atrial fibrillation quality of life questionnaire (AFQLQ). Jpn J Electrocardiol.

[14] Bazett HC (1920). An analysis of the time relations of electrocardiograms. Heart.

[15] Seki N, Nagao T, Komori K, Suzuki H (1988). Alpha and beta adrenoceptor blocking action of carvedilol in the canine mesenteric artery and vein. J Pharmacol Exp Ther.

[16] Cargnoni A, Ceconi C, Bernocchi P, Boraso A, Parrinello G, Curello S, Ferrari R (2000). Reduction of oxidative stress by carvedilol: role in maintenance of ischaemic myocardium viability. Cardiovasc Res.

[17] Palazzuoli A, Calabria P, Verzuri MS, Auteri A (2002). Carvedilol: something else than a simple betablocker?. Eur Rev Med Pharmacol Sci.

[18] Kawai K, Qin F, Shite J, Mao W, Fukuoka S, Liang CS (2004). Importance of antioxidant and antiapoptotic effects of β-receptor blockers in heart failure therapy. Am J Physiol Heart Circ Physiol.

[19] Poole-Wilson PA, Swedberg K, Cleland JG, Di Lenarda A, Hanrath P, Komajda M, Lubsen J (2003). Comparison of carvedilol and metoprolol on clinical outcomes in patients with chronic heart failure in the Carvedilol Or Metoprolol European Trial (COMET): randomised controlled trial. Lancet.

[20] Packer M, Coats AJ, Fowler MB, Katus HA, Krum H, Mohacsi P, Rouleau JL (2001). Effect of carvedilol on survival in severe chronic heart failure. N Engl J Med.

[21] Packer M, Bristow MR, Cohn JN, Colucci WS, Fowler MB, Gilbert EM, Shusterman NH (1996). The effect of carvedilol on morbidity and mortality in patients with chronic heart failure. U.S. Carvedilol Heart Failure Study Group. N Engl J Med.

[22] Hori M, Sasayama S, Kitabatake A, Toyo-oka T, Handa S, Yokoyama M, Matsuzaki M (2004). Low-dose carvedilol improves left ventricular function and reduces cardiovascular hospitalization in Japanese patients with chronic heart failure: the Multicenter Carvedilol Heart Failure Dose Assessment (MUCHA) trial. Am Heart J.

[23] Stroe AF, Gheorghiade M (2004). Carvedilol: β-blockade and beyond. Rev Cardiovasc Med.

